# A Statistical Assessment of Drilling Effects on Glass Fiber-Reinforced Polymeric Composites

**DOI:** 10.3390/ma17225631

**Published:** 2024-11-18

**Authors:** Ana Martins, Alda Carvalho, Ivo M. F. Bragança, Inês C. J. Barbosa, Joaquim Infante Barbosa, Maria A. R. Loja

**Affiliations:** 1 Centro de Investigação em Modelação e Otimização de Sistemas Multifuncionais (CIMOSM), Instituto Superior de Engenharia de Lisboa (ISEL/IPL), Av. Conselheiro Emídio Navarro 1, 1959-007 Lisboa, Portugal; ana.martins@isel.pt (A.M.); alda.carvalho@uab.pt (A.C.); ivo.braganca@isel.pt (I.M.F.B.); ines.barbosa@isel.pt (I.C.J.B.); joaquim.barbosa@isel.pt (J.I.B.); 2 Research Centre for Mathematics and Applications (CIMA), Instituto Superior de Engenharia de Lisboa (ISEL/IPL), Av. Conselheiro Emídio Navarro 1, 1959-007 Lisboa, Portugal; 3 DCeT, Universidade Aberta, Palácio Ceia, Rua da Escola Politécnica, n.° 147, 1269-001 Lisboa, Portugal; 4 CEMAPRE/ISEG Research, ULisboa, Rua Quelhas, 6, 1200-781 Lisboa, Portugal; 5 Instituto de Engenharia Mecânica (IDMEC), IST-Instituto Superior Técnico, Universidade de Lisboa, Av. Rovisco Pais, 1, 1049-001 Lisboa, Portugal

**Keywords:** composite materials, drilling, delamination characterization, thermographic characterization, statistical assessment, ANOVA

## Abstract

Fiber-reinforced composites are extensively used in many components and structures in various industry sectors, and the need to connect and assemble such types of components may require drilling operations. Although drilling is a common machining process; when dealing with fiber-reinforced composite materials, additional and specific problems may arise that can com-promise mechanical integrity. So, the main goal of this work is to assess how various input variables impact two main outcomes in the drilling process: the exit-adjusted delamination factor and the maximum temperature on the bottom surface where the drilling tool exits. The input variables include the type of drilling tools used, the operating speeds, and the thickness of the plates being drilled. By using Analysis of Variance (ANOVA), the analysis aims to identify which factors significantly influence damage and exit temperature. The results demonstrate that the influence of tools and drilling parameters is critical, and those selections impact the quality of the hole and the extent of the induced damage to the surrounding area. In concrete, considering the initially selected set of tools, the BZT03 tool does not lead to high-quality holes when drilling medium- and high-thickness plates. In contrast, the Dagger tool shows potential to reduce exit hole damage while also lowering temperature.

## 1. Introduction

As the use of composite materials continues to grow, the need to connect parts made of these materials increases the interest in understanding the drilling damage that occurs when preparing those parts. Because of the heterogeneous characteristics of composite materials, the damage that occurs during drilling, such as delamination and tearing, as well as thermomechanical changes due to the drilling effects on the composite, deteriorate the quality of the composite [1].

Several studies regarding the drilling of Fiber-Reinforced Polymer (FRP) composites can be found, among them, reviews on drilling Carbon Fiber-Reinforced Polymers (CFRP). An extensive review of drilling on CFRP presents relevant information found on drilling mechanisms, thermomechanical responses, drilling-induced damage, and the effects of various process conditions. High cutting speeds and low feed rates were found to improve the hole quality of CFRPs. Also, this review indicates that developing suitable tool geometries/materials, and the optimization of cutting parameters effectively decreases the drilling damage of cut CFRP holes [2]. The type of matrix material highly influences the thrust force and torque in conventional dry drilling of CFRP composites, while higher cutting speeds result in lesser torques developed during drilling [3].

To study the influence of machining parameters on the delamination damage of Glass Fiber-Reinforced Polymers (GFRP) during drilling, several drilling processes were explored and it was found that delamination is most influenced by feed rate, tool material, and cutting speed in conventional machining, while vibration assisted drilling and ultrasonic assisted drilling are more appropriate for drilling of GFRP [4].

As delamination is important damage resulting from drilling FRPs, many authors have investigated its mechanism and contributing factors [5,6,7,8,9,10,11,12]. In a review focused on delamination quantification and measurement techniques, several delamination measurement methods along with their advantages and drawbacks are compared and discussed [8]. The more accurate measurement techniques are X-ray radiography and computerized tomography, but are used less because of the high initial cost, the need for a secured area for inspection, and a high sample preparation time. Although with a lower accuracy, microscopy is the most generally used method due to its simplicity. Higher dimensionality of the delamination factor improves its accuracy but also increases computation time. The calculation of the delamination factor differs depending on the method used and many permutations and combinations are possible to define a more accurate delamination factor. Different models are used to predict drilling delamination, using different drilling parameters, such as cutting speed or cutting sequences [6,9].

The effect of tool material and geometry on the damage induced during drilling glass or carbon FRP composites is another interesting topic addressed by some authors [3,12,13,14,15]. Although extensive studies on the effect of cutting parameters and tool geometry on the quality of the hole have been made, the shearing of fiber-reinforced polymers needs a better understanding. Also, specific tools with special geometry need to be developed to achieve better performances [15]. In another article comparing different drilling tools on GFRPs, delamination was observed in the form of matrix debonding, uncut fibers, and fiber pull-out [7]. Results showed that the solid carbide tools had the best drilling performance for a low feed rate and a high speed, and high laminate thickness. Different drill types were also studied in drilling GFRP pipes and several tests were performed at a constant speed and different feed rates [16]. Thrust forces were measured and hole exit surface damage and borehole surface damage were examined with a digital microscope and scanning electron microscope, after the drilling operations. Damage is very much influenced by the tool geometry and feed rate, observing increased delamination for a conventional twist drill at lower feed rates, in comparison with a brad and spur drill, and a brad center drill. The latter generated less damage. New drilling tools are also presented in different works to improve hole quality, diminish delamination, and decrease overall damage on FRPs [14,17,18,19,20]. Hole quality is an important indicator of good drilling on FRPs, as it is related to less damage and smaller temperature-affected areas. Several studies seek to evaluate this indicator through different techniques such as studying bore quality factors’ influence on the progress of tool wear and the thrust force [21], the effect of speed, feed rate, and drill angle on hole quality [22], and the application of artificial neuron networks to assess the effects of drilling parameters on drilling temperature and hole quality [23].

New methods for predicting damage in FRPs have been devised. Several models were developed to predict the delamination factor [6,24,25]. The use of Deep Neural Networks resulted in very small errors in the predicted delamination factor and circularity, demonstrating that this technique is very suitable for predicting drilling damage on FRPs [6]. Other more simple modelling techniques have been proposed to predict delamination with great accuracy, such as AutoCAD Image Processing [24]. Other models focus on predicting the temperature distribution during the drilling process. In one study, a model to predict the temperature distribution during the drilling process of unidirectional CFRP is used to explore the influence of feed rate and spindle speed, and the results show that increasing the feed rate decreases the drilling temperature and the opposite occurs when increasing the spindle speed [26].

The variation of temperature during drilling of FRPs is an important parameter for the quality of the hole and, consequently, it is related to damage. Thus, many studies focus on thermal damage, cutting heat accumulation and tool wear extent during the drilling of FRP structures. Several strategies are used to diminish the wear of tool bits, such as variable feed rates and reducing the number of holes performed [27]. The surface integrity is also used as an indicator of the quality of the hole to assess the performance of special drills made for drilling CFRPs with less tool wear [28]. Many techniques are used to obtain information on temperature, such as infrared cameras [5,29,30] and thermocouples [26,31]. When considering the temperature-dependent material properties of CFRP laminates to study the problem of contact at the drill margin–borehole surface interface during dry drilling [31], it is known that the increase in temperature during dry drilling reduces the elastic modulus of the CFRP and causes thermal expansion of the drill. This causes significant contact length at the drilling margin and borehole surface interface, which, in turn, increases damage. The temperature increase also increases the thrust force and torque, indicating that low feed rates is disadvantageous to dry drilling because of the temperature rise due to inefficient material cutting.

The use of non-destructive testing methods for estimating damage in composite materials has been an established practice in research for years. However, several studies have noted the increase in research on non-destructive testing techniques, driven by the growing use of composites in industries like aerospace, automotive, and energy sectors, where ensuring the integrity of structures is critical [32]. Furthermore, advancements in computing power, sensor technology, and imaging techniques have made these methods more accessible and reliable, promoting broader applications [33]. Many of these methods, including ultrasonic testing, infrared thermography, shearography, and acoustic emission, have evolved, offering greater precision, faster data processing, and automated systems for inspecting composite structures [32,33,34,35,36,37,38].

In another complementary perspective, exploratory data analysis, although consisting of an important research area by itself, plays a very important role when linked to specific engineering problems, as it may provide important insights into the experimental and/or modelling approaches observations, identifying the components, parameters, that most contribute to explain the observed experimental or simulated responses. To illustrate this, one may refer to the work developed by some co-authors, in complementary areas. Carvalho et al. [39] investigated the variability in the static and dynamic response of fiber-reinforced composites, considering multivariable linear regression models, to characterize the contribution of each modelling parameter to the explanation of those variabilities. Focusing on another type of composite materials, Rosa et al. [40] analyzed how material and geometrical uncertainty may modify the foreseen deterministic response of a structure built from dual-phase functionally graded materials. In that work, the authors proposed the constitution of statistic models to allow their use as alternative prediction models for such structures under similar operating conditions. More recently Carvalho et al. [41] investigated the influence that the uncertainty associated with carbon nanotubes’ material and geometrical characteristics may have in the static behavior of functionally graded plates, where this gradient is dictated by the weight volume fraction of these nanoparticles. The study considered the constitution of multiple regression models which allow concluding on the influence of the characteristic parameters and also can be used as alternative prediction tools within the domain of the study.

In the present work, drilling parameters, such as the spindle speed, the feed rate, the GFRPs laminates’ thicknesses, and tool bits, are considered and their influence is assessed on a set of usual delamination factors that are often used as damage metrics, and on the thermally influenced area.

Regarding the authors’ knowledge, this work has an innovative character, considering the joint glass fiber composites’ drilling experimental work and the detailed statistical study performed upon the results achieved. This statistical approach enables an extensive analysis and corresponding conclusions regarding the engineering problem this work addresses, namely considering the influence of tool selection, material thicknesses on final part drill quality, as well as the importance of drilling parameters in minimizing undesirable effects.

The remainder of this manuscript presents the following structure: the second section presents the fundamental theoretical aspects required for the study’s development, followed by the third section where the case studies and the results achieved are presented and discussed and more specific conclusions are drawn. The last section refers to the conclusions of the present work.

## 2. Materials and Methods

### 2.1. Composites Plates and Experimental Setup Characterization

In the present work, four sets of glass fiber polymeric laminated plates were produced via a wet layup method: a set of plane laminates consisting of 10 layers of short glass fibers; and three sets of plane laminates made of fiberglass textile and epoxy resin, with 10, 20, and 30 layers, respectively. All sets had a quadrilateral configuration with a 250 mm edge. After the curing process, the plates were characterized and identified, as presented in Table 1.

Glass fiber was herein considered either in the form of fabric, produced with interwoven long fibers, or in the form of mat, which consists of short fibers randomly overlapped, nonwoven. These two types of fiber dispositions represent the most common alternatives used in glass fiber composite manufacturing.

Different plates were prepared as indicated in Table 1. The wet layup technique was used, with Sicomin’s SR1500 resin mixed with hardener SD2505 in a 3:1 ratio. The various layers were impregnated with the resin and cured at room temperature under compression between metal plates.

**Figure 1:**
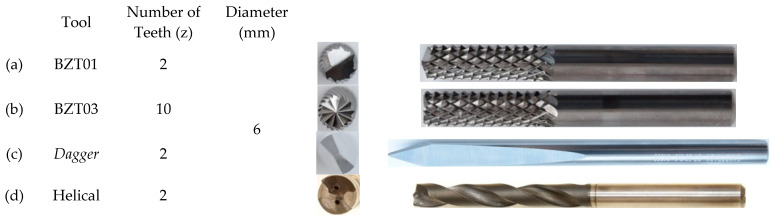
Cutting tools: (**a**) BZT01 Tool (Manufacturer BZT, Leopoldshöhe, Germany, Ref. 751080060F); (**b**) BZT03 Tool (Manufacturer BZT, Ref. 751070060F); (**c**) Dagger Tool (Manufacturer GANDTRACK, Oldham, UK, Ref. GT-50-6.0 63089); (**d**) Helicoidal Tool (Manufacturer SECO, Fagersta, Sweden, Ref. SD205A-6.0-32-6R1-C2).

Figure 1 presents the tools used in the drilling experiments.

As the material properties of GFRC are highly dependent on the fabrication method, and on the uncertainty of associated sources, thermographic analysis was used to find correlations between the material defects and the drilling outcome.

To perform the drilling operations, a specific support was developed to be adjusted to the computerized numerical control machine (CNC, Cincinnati Arrow 500 VMC, Cincinnati, OH, USA), and two thermographic cameras (ThermaVue CSI 735R INFRARED (Southampton, UK) and TROTEC ec060 (Heinsberg, Germany)) were used to record the operation from the upper and lower laminate surfaces’ perspectives.

**Figure 2:**
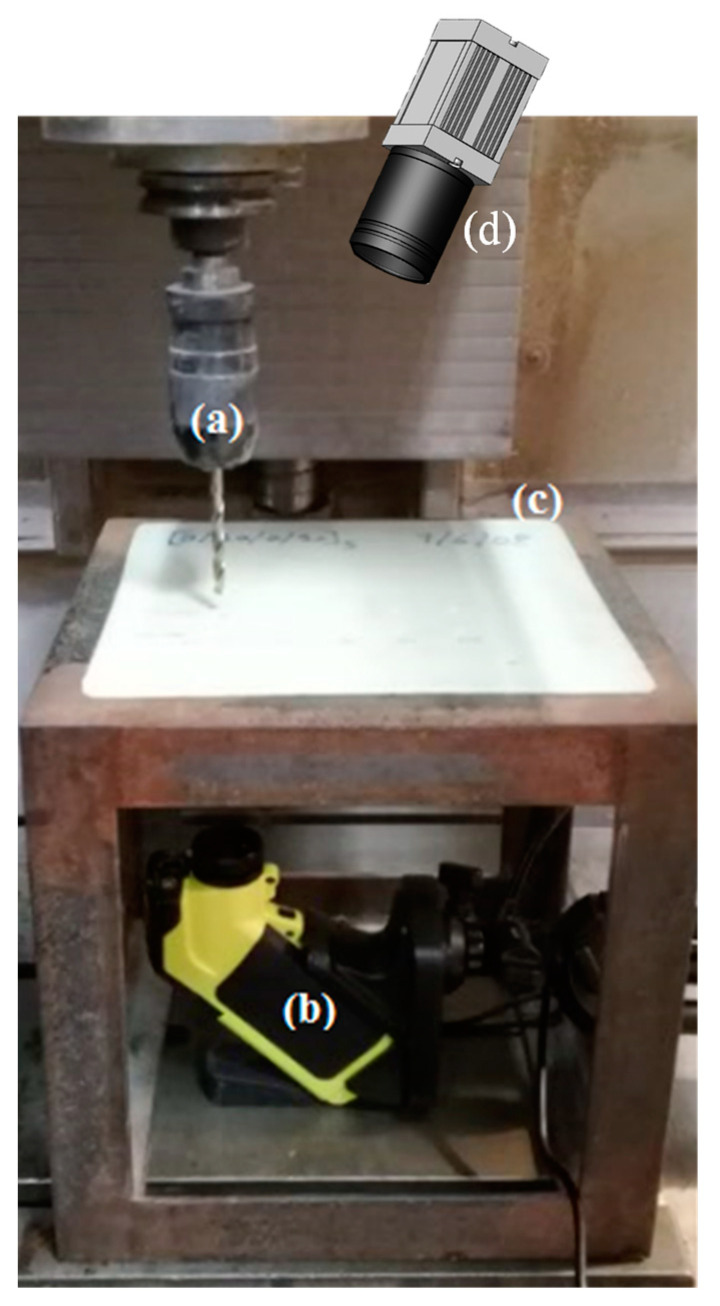
Experimental apparatus: (**a**) drilling tool; (**b**) lower thermographic camera; (**c**) plate; (**d**) upper thermographic camera.

The camera recording the lower surface of the laminate was aligned with the tool axis, while the upper surface camera had a slight deviation to this axis due to being put near the tool. The experimental apparatus is shown in Figure 2.

After conducting preliminary experimental tests for the thicker laminate using the BZT03 tool, the temperature range was verified to be within [23–250 °C].

### 2.2. Delamination Quantification Metrics

To perform the statistical assessment of the results, we considered the various affected areas, including those related to delamination induced by drilling and those associated with thermally affected regions in the laminate. The quantification of delaminated areas has been explored by various authors, who have employed different methods to assess the extent of the damage. One usual indicator is the damage factor Fd [42,43]; however, in this work, the adjusted delamination factor, $F_{daj}$, that considers the contribution of the fissure size and the damage area [44] is more extensively used:

(1) $$F_{daj}=F_{d}+\frac{A_{d}}{\left(A_{max}-A_{0}\right)}\left({F_{d}}^{2}-F_{d}\right)$$

where $A_{d}$ is the delaminated area, $A_{max}$ and $A_{0}$ are, respectively, the areas corresponding to the maximum damage diameter, $D_{max}$, and to the hole diameter, $D_{0}$. An alternative assessment of the delaminated area was proposed by Ahn et al. [45], which considers the existing proportionality between the image areas and the corresponding number of pixels:

(2) $$F_{da}=1+\frac{N_{p}\left(A_{d}\right)}{N_{p}\left(A_{0}\right)}$$

where $N_{p}\left(A_{d}\right)$ is the number of pixels of the delaminated area, and $N_{p}\left(A_{0}\right)$ is the number of pixels corresponding to the hole area. The maximum delamination diameter $D_{max}$ and the delaminated area $A_{d}$ are obtained as:

(3) $$D_{max}=D_{0}\frac{N_{p}\left(D_{max}\right)}{N_{p}\left(D_{0}\right)}$$

where $N_{p}\left(D_{max}\right)$ is the number of pixels of the maximum diameter area, and $D_{0}$ is the hole diameter. This last calculation of the damaged area, $F_{da}$, has proven to be more reliable than the damage factor, $F_{d}$, as the latter is not sensitive to the in-plane shape of the delamination profile. The studies conducted take these different measurements into account to characterize the results of the experimental drilling tests.

To identify the delaminated and thermally affected regions resulting from drilling operations, scans were performed on both the upper and lower surfaces of the laminate. The entire drilling process was also recorded using thermographic cameras. The captured digital data were subsequently processed with the open-source image processing software Fiji (ImageJ 1.53q, public domain (Bethesda, MD, USA) [46], the images were examined, and the extent of the damage was quantified.

**Figure 3:**
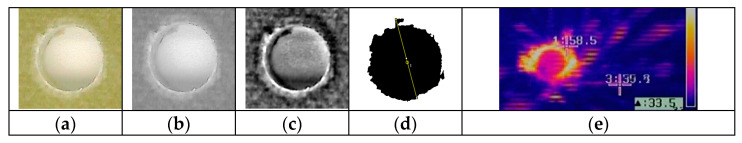
Procedure to evaluate the delaminated and thermally area affected by drilling: (**a**) original photograph; (**b**) binary image; (**c**) image after FFT bandpass filtering; (**d**) diameter measured after thresholding. (**e**) Thermographic image from lower camera.

After calibrating the initial image dimensions, Figure 3a, the photograph was converted to binary grayscale to isolate the damaged areas, Figure 3b. The FFT bandpass filter corrected shadows and smoothed the image, and the threshold tool was applied using the iterative procedure known as the IsoData algorithm [47], as seen in Figure 3c. The resulting binary image was used to determine the maximum damage diameter through a line that passes through the hole’s center, Figure 3d.

In addition to the discrete temperature data provided by the thermographic cameras at specific points (see crosses in Figure 3e), a complementary methodology based on the RGB color system was employed to characterize the maximum temperature reached during the drilling process. The color scale of the images was also taken into account. This procedure ensures that the maximum temperature in the selected image is measured, as it may be somewhat distant from the points marked by the crosses. However, the presence of uncut material, fibers, expelled material, as well as the determination of the frame considered to reach the maximum value, can influence the determined value.

### 2.3. Statistical Analysis Methodology

One way to compare a variable across different categories is through a graphical representation using multiple boxplots [48]. This graphical tool shows the distributions side by side, allowing for a clear comparison of their medians, ranges, and variability.

An important graphical tool for visualizing a multivariate dataset is a composition of several plots, referred to here as a matrix plot. This plot allows for the analysis of both the individual distributions and the different correlations between variables. In this type of plot, the individual distribution of the variables under analysis is shown along the diagonal of the matrix. An empirical density line is added, providing a suggestion of the appropriate probability model for each variable. This type of analysis is important for assessing the assumptions often required for inferential purposes. Additionally, matrix plots allow for the analysis of correlations between pairs of variables. Below the diagonal, scatter plots are displayed, while above it, the values of Pearson’s linear correlation coefficient are shown [48].

Analysis of Variance (ANOVA) is a statistical method used to determine if there are statistically significant differences between the means of three or more independent groups [48]. ANOVA tests the null hypothesis that all group means are equal,

$$H_{0}:\;\mu_{1}=\mu_{2}=\cdots =\mu_{p}\;\;\;\;\;\;\;\;vs\;\;\;\;\;\;\;\;H_{1}:\;\exists \;\mu_{i}\neq \mu_{j}\;$$

and if this hypothesis is rejected, it indicates that at least one group differs from the others. However, ANOVA does not specify which groups differ from each other, so post-hoc tests, such as Tukey’s Honestly Significant Difference (HSD) test, are often used to perform pairwise comparisons between group means. Tukey’s HSD test controls for Type I errors by adjusting the significance level in multiple comparisons, ensuring a more reliable identification of specific differences between group means. It is possible to visualize these differences through the graphical representation of confidence intervals for the difference between means [48].

ANOVA relies on several key assumptions to ensure the validity of its results [48]. It assumes that the data in each group follow a normal distribution. This assumption can be checked using normality tests such as Shapiro–Wilk [49]. Also, it assumes the independence of observations, meaning that the data points in each group are independent of each other, which is often guaranteed by proper experimental design. All ANOVA results presented in this article meet these assumptions with a significance level of 1%.

When the assumptions of a parametric test are violated, a non-parametric approach is applied, such as the Kruskal–Wallis test [50]. This method is used to compare three or more independent groups and to assess whether there are statistically significant differences in their medians.

## 3. Results and Discussion

As an introductory note in this section and with no prejudice to more detailed descriptions that will be provided in each sub-section, it is considered adequate to make an overall brief presentation of the data analysis flow which was supported by intermediate conclusions.

Hence, due to some experimental limitations, and issues encountered during the experimental tests, the statistical analysis was conducted sequentially.

In the first phase, the aim was to determine if there were differences among the four tools used in terms of damage indicators or temperature within the considered range of values (spindle speed of 5300 rpm and feed rate of 640 mm/min). This initial case allowed to identify high correlations in the output variable values at the entrance and exit. These values remained similar throughout the tests, which led us to focus on the exit-adjusted delamination factor, the variable Fdaj_out, for damage analysis. Furthermore, based on the results obtained, it was decided to continue the study using only the BZT03 and the Dagger tools, as they represent two very different performances in terms of damage.

In the second phase, given the distinct nature of the two tools, a study of both tools was conducted in parallel. The aim of this study was to understand the influence of using low, medium, or high ranges in a combination of speed and feed rate on the damage factor. In the third phase, different thickness values were then analyzed.

In the end, a discussion was elaborated and provided for each of the two tools (BZT03 and Dagger) regarding the relationship between damage and temperature across the different categorical dimensions used (feed rate and thickness).

### 3.1. Variables Characteristics

The input variables considered in the present work essentially comprise the drilling tools used, the operating speeds, and the thickness of the plates to be drilled.

**Figure 4:**
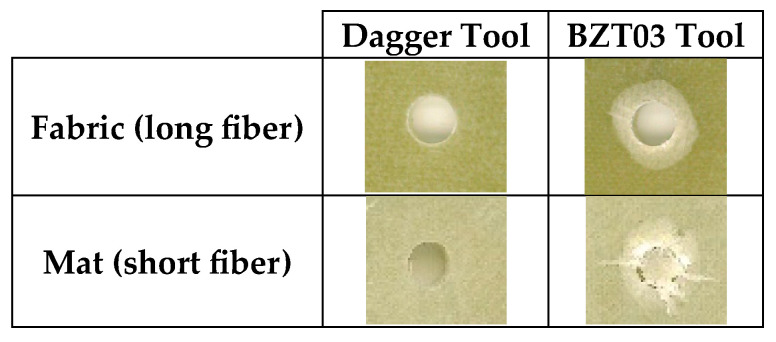
Holes’ exits performed by Dagger and BZT03 in plates reinforced with small or long fibers (spindle speed 5300 rpm; feed rate 640 mm/min).

The drilling of the samples, considering the different variables, was performed and the effect on the samples was registered, as shown in Figure 4 for one of the samples. Despite being by far the drill that makes the best holes and causes significantly less damage, the Dagger tool sometimes leaves some fibers uncut at the exit.

After conducting some exploratory tests and observing minimal variation in the effect of drilling parameters on damage of different plates with different fiberglass textile, it was decided to evaluate only the holes made in the long-fiber plates. This choice helped reduce the variation in measured exit damage, often caused by factors like a single fiber being pulled out rather than delamination between layers.

The output variables are coefficients calculated according to damage characterization indicators, and the maximum temperature at the bottom surface where the drilling tool exits the plate. The input variables and data associated as well as the output variables and corresponding acronyms are presented in Table 2.

Considering the characteristics of the input variables, they were all considered categorical variables. From the experimental tests developed, 76 (see Appendix A) were considered valid, being those summarized in Table 3.

Concerning the information in Table 3, it is relevant to note that the column corresponding to the thickness variable, besides containing the values themselves, also contains the indication of a qualitative classification {low, medium, high}. This is because, for a set of situations where the thickness assumes the values of 2.88 mm, 2.95 mm, and 3 mm, this was not intentional and corresponds to uncertainties in the manufacturing process. So, this set of thicknesses is considered to pertain to the same class of thinner plates.

Cutting tools with the same diameter but different characteristics were selected to evaluate the influence of the tools’ geometry. Some of the selected tools resemble milling cutters, while others are more similar to traditional drill bits. This selection aimed to analyze a wider range of tool types for drilling holes in glass fiber epoxy plates.

### 3.2. Case 1: Assessing the Influence of Input Variables on the Exit-Adjusted Delamination Factor for an Initial Set of Drilling Tools

In the first stage of this work, we began by considering five experimental tests for each of the four tools under study. All the drilling tools were tested under the same conditions (spindle speed of 5300 rpm and feed rate of 640 mm/min) on plates with a uniform thickness of 2.88 mm.

In this preliminary case, it is also important to say that the output variables at the tool entrance on the top surface of the plate were also initially registered, but they were shown to be less significant when compared to the results obtained at the exit. So, only the exit output variables will be considered.

**Figure 5:**
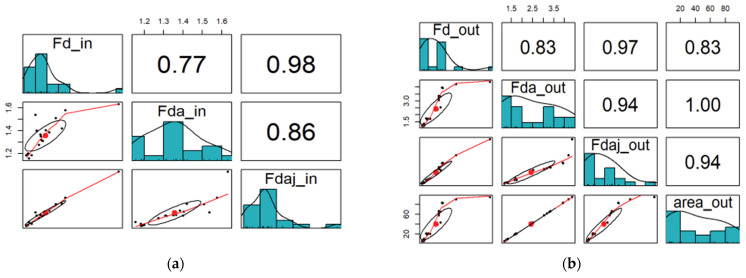
Matrix plots for the output variables; (**a**) entrance, and (**b**) exit.

The correlation coefficients between the different variables can be observed in Figure 5, where the matrix plots corresponding to the entrance and exit output variables are presented. The red line corresponds to a smooth curve called Locally Estimated Scatterplot Smoothing (LOESS). This method is useful for visualization and is constructed by fitting multiple quadratic (or possibly linear) regression lines as a moving window passes along the x-axis. The black dots are the data and the red one is the corresponding mass center.

Considering the results in the matrix plot of the exit output variables, it is visible that the variables are strongly correlated, namely the exit delamination factor (Fd_out) and the exit-adjusted delamination factor (Fdaj_out), the exit-delamination area factor (Fda_out), and the exit-delaminated area (area_out). This is an expected result considering how these metrics are obtained. Because of this, we have decided to keep only one of these metrics to characterize the damage, being this metric, the Fdaj_out variable. So, from this point on, only the exit output variables will be considered, more specifically the exit-adjusted delamination factor.

#### 3.2.1. Influence of a Tool on the Exit-Adjusted Delamination Factor at the Tool’s Exit

**Figure 6:**
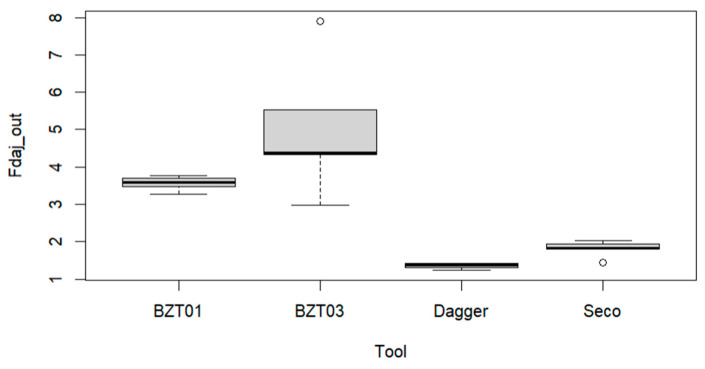
Multiple boxplots for the exit-adjusted delamination factor as a function of the tools BZT01, BZT03, Dagger, and Seco.

Following the previous analysis, it was important to understand which tool would be more adequate for the drilling operation. If one builds the boxplots of the exit-adjusted delamination factor for each tool, one obtains the representations in Figure 6.

From Figure 6, we conclude that there is a significant difference among the Fdaj_out variable mean values associated with each tool. It is possible to verify that the Dagger and Seco tools enable drilling with a lower exit damage value for the plates. In contrast, BZT tools exhibit not only a higher exit-adjusted delamination factor but also higher uncertainty. So, Dagger tools offer a more consistent drilling performance with greater uniformity and quality, providing superior control over the process.

Table 4 presents the ANOVA results from the comparison of the Fadj_out mean values for the four tools, allowing for the conclusion that there is a significant difference among them. The first column lists the different sources of variability (Tool, Error, Total), indicating where the variation in the data originates. The second column shows the degrees of freedom (df), representing the number of independent observations that can vary for each source of variation. The third column contains the Sum of Squares (SS), which measures the total variability attributed to each source and quantifies how much variation is explained by each factor. The fourth column presents the Mean Square (MS), calculated by dividing the Sum of Squares by the degrees of freedom; this gives the average variation for each source of variability (MS = SS/df). The fifth column provides the F-Statistic, the ratio of the mean square between groups to the mean square within groups, which tests whether the variability between groups is greater than that within groups, indicating a significant effect. Finally, the last column displays the p-value, which represents the probability of obtaining a result at least as extreme as the observed one, assuming that the null hypothesis (the hypothesis that there is no difference) is true. A low p-value suggests that the differences between groups are statistically significant. To aid in interpreting the results, a code is added to the *p*-value to indicate whether the statistical significance is below 0.001 or at 0.01.

**Figure 7:**
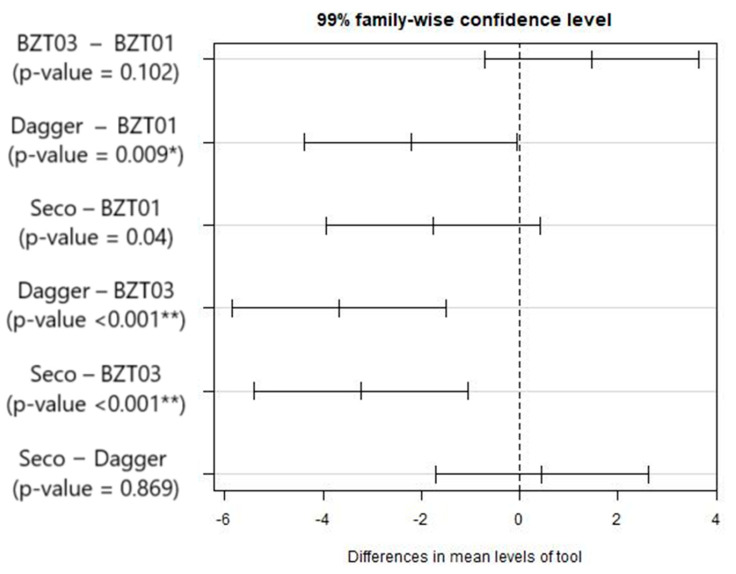
Results from the multiple comparisons test, between pairs of tools: confidence intervals for mean differences. Significance codes: <0.001 ‘**’ 0.01 ‘*’.

Given that the equality of the mean values has been rejected, it is appropriate to examine the differences between each pair. So, a multiple comparisons test (Tukey test) was performed. The results obtained are presented in Figure 7.

Considering the results in Figure 7, it is possible to conclude that the BZT01 and BZT03 tools do not differ significantly, the same applying to the Dagger and Seco pair and the Seco and BZT01 pair. Supported by these results, we proceeded with further tests considering only the BZT03 and Dagger tools, which is in agreement with what was already observed in Figure 6.

#### 3.2.2. Influence of Spindle Speed and Feed Rate on the Exit-Adjusted Delamination Factor at the Tool’s Exit

The experimental data were increased to evaluate the influence of the spindle speed and the feed rate on the exit-adjusted delamination factor at the tool’s exit, as shown in Table 5.

**Figure 8:**
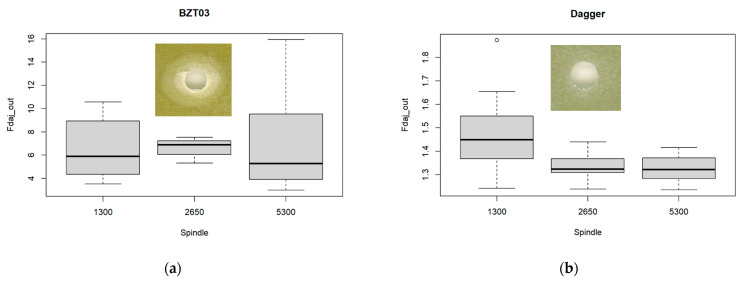
Multiple boxplots for the exit-adjusted delamination factor at the tool’s exit as a function of the spindle speed for the (**a**) BZT03, and (**b**) Dagger tools.

The analysis of the boxplots in Figure 8, relating the exit-adjusted delamination factor at the tool’s exit with the spindle speed for each one of the tools, raises doubts regarding the hypothesis of equality of the mean value of the exit-adjusted delamination factor in the three ranges of the spindle speed considered (low, medium, and high).

To confirm, an ANOVA was performed and the p-values obtained from the ANOVA conducted for the BZT03 and Dagger tools (Table 6 and Table 7) were respectively 0.99 and 0.02. Considering a significant level of 1%, there was no significant difference in the mean value of the exit-adjusted delamination factor in the three ranges of the spindle speed considered (low, medium, and high).

**Figure 9:**
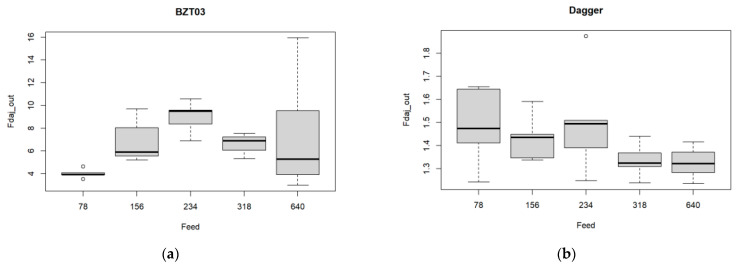
Multiple boxplots for the exit-adjusted delamination factor at the tool’s exit as a function of the feed rate for the (**a**) BZT03, and (**b**) Dagger tools.

A similar approach was conducted regarding the feed rate. The corresponding boxplots are presented in Figure 9.

Although there are differences in Figure 9, they are not significant. The ANOVA results (Table 8 and Table 9) indicate that when considering a significant level of 1%, there are no significant differences in Fdaj_out, for all the feed rate values considering both the Dagger and the BZT 03 tools.

### 3.3. Case 2: Assessing the Maximum Temperature at the Tool’s Exit as a Function of the Spindle Speed and the Feed Rate for the BZT03 and Dagger Tools

In the sequel of the previous case study, where two tools (BZT03 and Dagger) were selected considering their differentiated characteristics regarding the delamination assessment, the present case study proceeds with those same tools to assess how the input parameters may influence the maximum temperature achieved at the tool’s exit location, in the bottom surface of the plates.

The experiment characteristics used to assess the influence of the spindle speed and the feed rate on the maximum temperature at the tool’s exit were already presented in Table 3.

**Figure 10:**
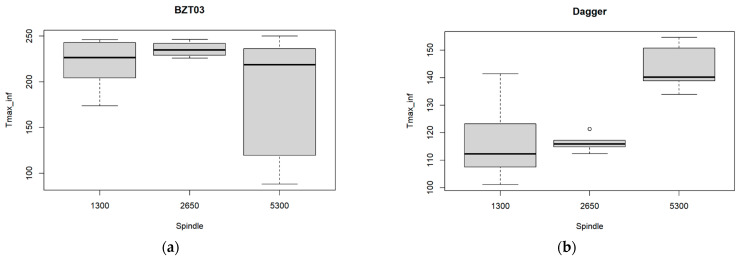
Multiple boxplots for the maximum temperature at the tool’s exit as a function of the spindle speed for the (**a**) BZT03 and (**b**) Dagger tools.

From this experimental data obtained, we have built the boxplots in Figure 10, where the exit-adjusted delamination factor at the tool’s exit is related to the three spindle speed values tested for each one of the tools. Those boxplots in Figure 10a suggest that for the BZT03 tool, some differences in the mean value of the maximum temperature at the tool’s exit, for the three spindle speed values. However, the ANOVA confirms that they are not significant (*p*-value = 0.03) (Table 10).

However, Figure 10b and Table 11 show that for the Dagger tool, there exists a significant difference among the three spindle speed values (ANOVA *p*-value < 0.001). The temperature for the higher speed significantly differs from the mean temperature for the other two spindle speed values.

**Figure 11:**
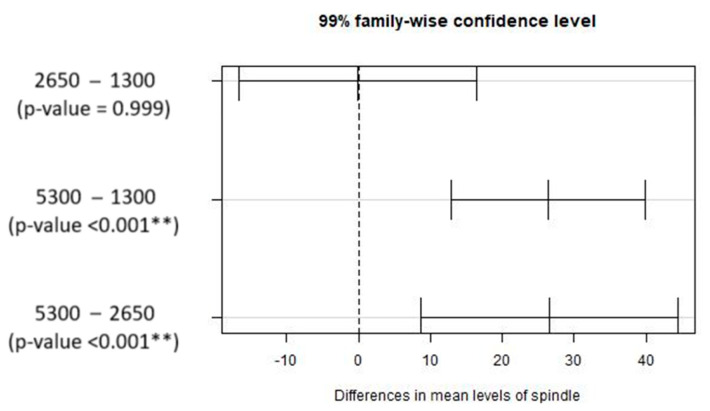
Results from the multiple comparisons test for pairs of spindle speed: confidence intervals for mean differences. Significance codes: <0.001 ‘**’ 0.01 ‘*’.

Figure 11 depicts the multiple comparison tests between the pairs of spindle speed values considered in the experiments. It can be concluded that a significant difference exists for the cases: 5300–1300 rpm and 5300–2650 rpm. It can be inferred that using the maximum spindle rotation speed in this study will lead to significant differences when compared to intermediate or lower speeds, making it more relevant to evaluate the extreme values.

**Figure 12:**
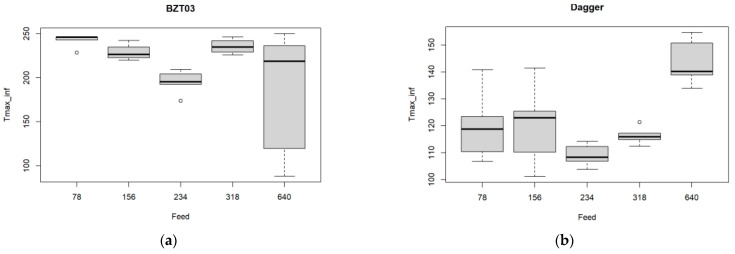
Multiple boxplots for the maximum temperature at the tool’s exit as a function of the feed rate for the (**a**) BZT03 and (**b**) Dagger tools.

The boxplots for the maximum temperature at the tool’s exit at the bottom of the plates, as a function of the feed rate, are presented in Figure 12.

Regarding the BZT 03 tool boxplots presented in Figure 12a, the null hypotheses of the Shapiro normality test were rejected, so the non-parametric Kruskal–Wallis test was used.

The mean temperature value is significantly different for the five feed rate values (*p*-value < 0.001). The multiple comparison tests allow us to conclude that there exists only a significant difference for the cases 78–234 mm/min.

For the Dagger tool results presented in Figure 12b one observes that the mean temperature value is significantly different for the five feed rate values considered (ANOVA *p*-value < 0.001, see Table 12).

**Figure 13:**
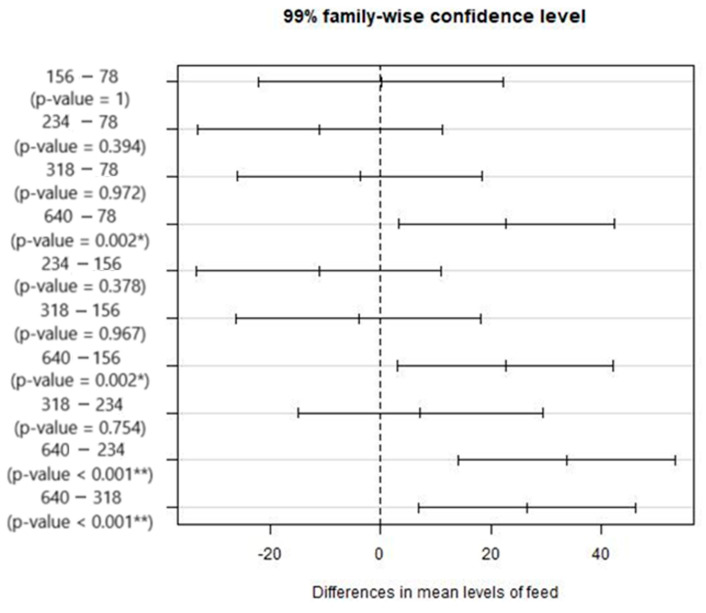
Results from the multiple comparisons test between pairs of feed rate values: confidence intervals for mean differences. Significance codes: <0.001 ‘**’ 0.01 ‘*’.

The multiple comparison tests (Figure 13) show that a significant difference exists for the cases: 78–640 mm/min, 156–640 mm/min, 234–640 mm/min, and 318–640 mm/min. Concerning Shapiro normality test, it was verified.

### 3.4. Case 3: Influence of Plates’ Thickness on the Exit-Adjusted Delamination Factor and the Maximum Temperature at the Tool’s Exit

To analyze the influence of the plates’ thickness on the exit-adjusted delamination factor and on the maximum temperature at the tool’s exit neighborhood, a few more experiments were conducted using only the BZT 03 tool. Table 13 summarizes these experimental characteristics.

**Figure 14:**
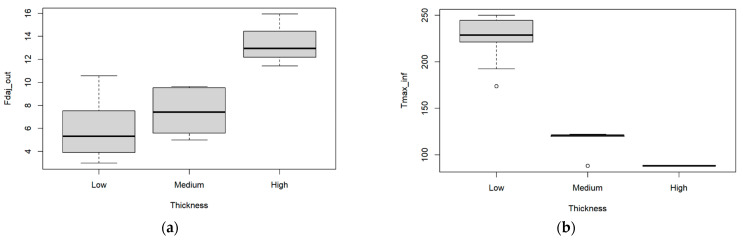
Multiple boxplots for the (**a**) exit-adjusted delamination factor, and for the (**b**) maximum temperature at the BZT3 tool’s exit as a function of three classes of thicknesses—low, medium, and high.

The boxplots corresponding to the results obtained from these experimental tests are presented in Figure 14.

**Figure 15:**
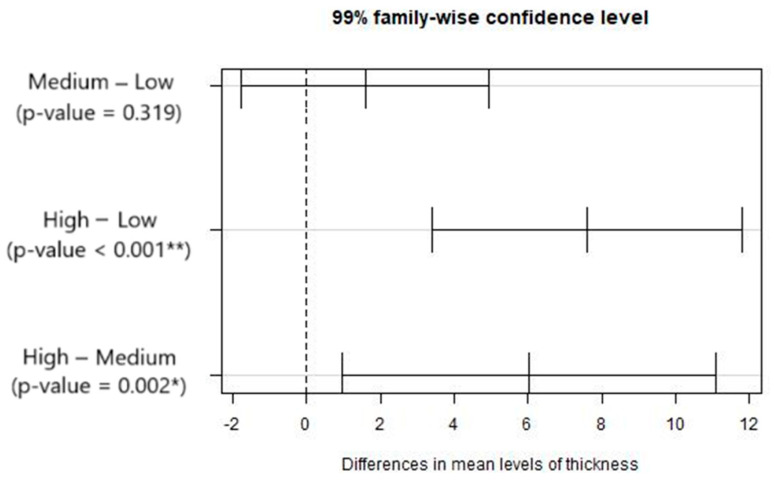
Results from the multiple comparisons test between pairs of mean levels of thicknesses: confidence intervals for mean differences. Significance codes: <0.001 ‘**’ 0.01 ‘*’.

The results from the ANOVA (Table 14) and multiple comparison tests (Figure 15) indicate that the mean of Fdaj_out is significantly different for the high and medium thickness as for the high and low thickness. An inverse relation is observed between the damage factor and the maximum temperature as the plate thickness varies. The damage values increase with greater thicknesses, even though the temperature on the lower side of the plate decreases. This may be caused by the tool’s geometry, which does not promote good chip removal, potentially leading to poorer cutting performance in the final stage. This may result in greater compressive forces and contribute to greater delamination of the composite.

However, regarding the maximum temperature at the tool’s exit, the null hypothesis of the Shapiro normality test was rejected, so we resorted to the non-parametric Kruskal–Wallis test. This latter test indicates that the mean of the output variable, Tmax_inf, is significantly different for the three thickness classes. The corresponding non-parametric multiple comparison tests indicate that the mean of Tmax_inf is significantly different for the low and medium thicknesses as well as for high and low thicknesses.

### 3.5. Case 4: The Relations Among Temperature, Exit-Adjusted Delamination Factor, and Other Input Variables, for the BZT03 and Dagger Tools

This final sub-section devoted to the presentation and discussion of the results is intended to analyze if and how the exit-adjusted delamination factor (Fdaj_out) and the maximum temperature (Tmax_inf) at the bottom surface are related.

To avoid redundancy, the presentation of results is limited to some sufficiently illustrative studies to demonstrate the behaviors’ trends. The BZT03 and Dagger tools were used separately to analyze the correlation between the two output variables (Tmas_inf and Fdaj_out).

#### 3.5.1. Influence of Plates’ Thicknesses and Spindle Speed on the Exit-Adjusted Delamination Factor and Maximum Temperature at the Tool’s Exit for the BZT03 Tool

**Figure 16:**
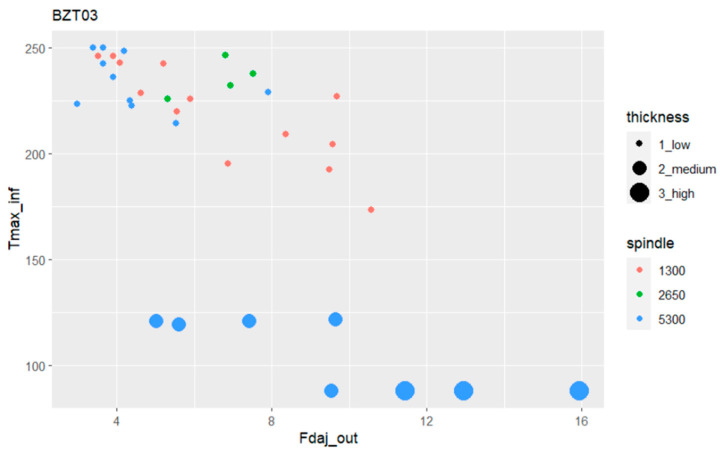
Bubble plot relating the maximum temperature with the exit-adjusted delamination factor, the thickness class, and spindle speed for the BZT03 tool.

According to the analysis performed in Section 3.3, it was possible to conclude that the plates’ thickness classes (low, medium, and high) differently affect the exit-adjusted delamination factor and the maximum temperature at the tool’s exit. In the present study, we aim to introduce an additional input variable into this analysis, namely the spindle speed. The relations among this wider set of variables are presented in Figure 16.

The bubble plot in Figure 16 illustrates very clearly that for medium- to high-thickness plates, we achieved the highest values for the exit-adjusted delamination factor, although for lower maximum temperatures. For the thickest plates (“high” class of thicknesses) the lowest temperatures were achieved although presenting the highest adjusted delamination factors.

Figure 16 also shows that when dealing with thinner plates in the so-called “low” class, although attaining the highest temperatures, the use of the highest spindle speed (5300 rpm) provides the lowest exit-adjusted delamination factor values. For lower values of spindle speed, namely for the lower one, the exit-adjusted delamination factor starts to increase despite presenting a greater dispersion in the delamination factor.

Since the thicker plates, medium and high, show exit-adjusted delamination factor values assessed as too high to be considered successfully executed, these variations will not be taken into account in the upcoming comparisons.

#### 3.5.2. Influence of Spindle Speed and Feed Rate on the Exit-Adjusted Delamination Factor and Maximum Temperature at the Tool’s Exit, for the BZT03 Tool

Regarding the BZT03 tool, the Pearson’s correlation coefficient between the variables (Tmax_inf) and (Fdaj_out) is −0.730, so there is an inverse correlation.

**Figure 17:**
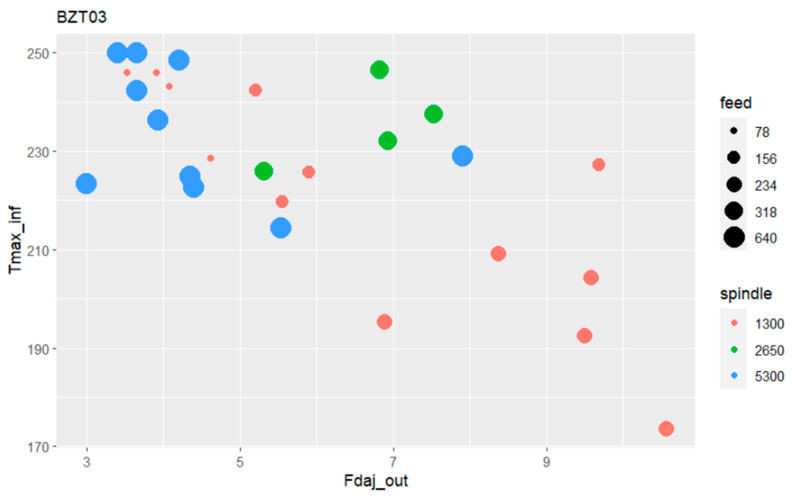
Bubble plot relating the maximum temperature with the exit-adjusted delamination factor and the feed rate and spindle speed for BZT03 tool.

Figure 17 presents for the BZT03 tool a bubble plot with the relative influence of the input variables, spindle speed, and feed rate in the exit-adjusted delamination factor and the maximum temperature at the tool’s exit in the plates’ bottom surfaces.

As the bubble plot in Figure 17 illustrates, when the BZT03 tool operates at 5300 rpm with a feed rate of 640 mm/min, higher values of temperature at the tool’s exit (near the maximum value for the camera) are attained, although with a lower exit-adjusted delamination factor. For the spindle speed of 2650 rpm the temperatures are similarly high although the damage presents in general a higher metric value.

The lower spindle speed (1300 rpm) when combined with the lowest feed rate (78 mm/min) yields very high-temperature values although the lowest damage values; however, when the feed rate is augmented until 234 mm/min, the temperature progressively diminishes and the exit-adjusted delamination factor increases, assuming values within the range of 6.5 to 11. Even considering only the tests that used the same feed per tooth with the BZT03 tool, it can be stated that as the drilling speed increases, the damage at the exit of the hole decreases, despite the temperature rising.

#### 3.5.3. Influence of Spindle Speed and Feed Rate Speed on the Exit-Adjusted Delamination Factor and Maximum Temperature at the Tool’s Exit for the Dagger Tool

After a more detailed study in the previous sub-sections regarding the BZT03 tool, this sub-section aims to focus on the Dagger tool, to identify the main influences and relations among the output variables and the input variables associated with the drilling operating parameters.

Regarding the Dagger tool, the Pearson’s correlation coefficient between the maximum temperature and the exit-adjusted delamination factor at the tool’s exit is -0.367, in this case also an inverse correlation, although less strong when compared with the results obtained for the BZT03 tool.

**Figure 18:**
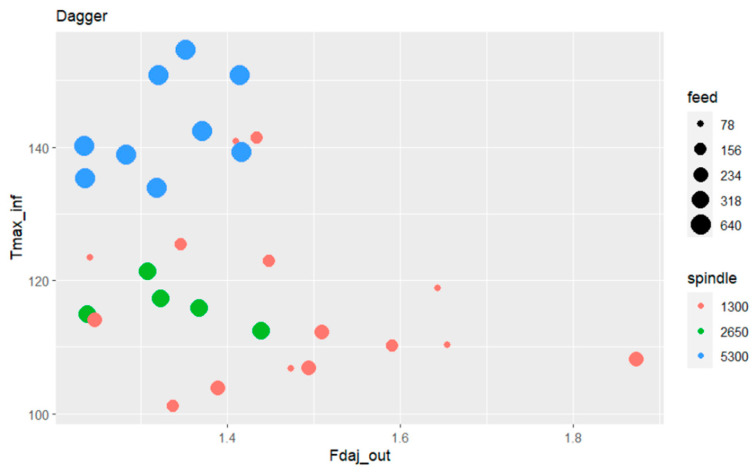
Bubble plot relating the maximum temperature with the exit-adjusted delamination factor, the feed rate and spindle speed for Dagger tool.

Figure 18 presents the bubble plot where the relations among these output and input variables can be observed.

With the Dagger tool it is possible to understand that overall, regardless of the spindle speed used, the exit-adjusted delamination factor is significantly minor compared with those presented when the drilling tool is the BZT03. It is also visible that the highest values of maximum temperature appear for the higher spindle speed values, although with a minor magnitude when compared with the values attained with the BZT03 tool.

Variation in the machining parameters appears to have a limited influence on the damage at the tool exit, despite some changes in temperature. Thus, for these ranges and this tool, there does not seem to be a significant correlation between these two outputs.

## 4. Conclusions

In this study, an assessment of damage and temperature in holes drilled in glass fiber composite plates has been successfully conducted using various statistical tools. An experimental setup was established to evaluate these parameters, and the statistical tools proved essential for interpreting the numerous tests performed. This approach helped to identify differences between the various tests and excluded those that do not significantly vary.

As an overall conclusion, the results show that the tool selection and the composite thickness show a great impact on the output parameters analyzed.

The influence of tools and drilling parameters is critical, as these selections directly impact the quality of the holes and the extent of damage at the hole exit. The determination of operational parameters to minimize undesirable effects is strongly correlated with the type of drill selected, indicating that the optimal parameters for one tool may differ significantly from those for another.

The Tukey test revealed that the Dagger and Seco tools resulted in lower exit damage values for the plates, while the BZT tools displayed a higher exit-adjusted delamination factor and greater variability. In contrast, the Dagger tools provided more consistent drilling performance, with greater uniformity and control, leading to superior overall quality in the drilling process. Also, the results indicate no significant difference between the BZT01 and BZT03 tools, as well as between the Dagger and Seco tools, and similarly between the Seco and BZT01 tools.

The BZT03 tool does not allow the manufacture of good-quality holes when drilling medium and high thickness plates. An inverse relation is observed between the damage factor and maximum temperature as plate thickness changes. Damage increases with thicker plates, while temperatures on the lower side decrease. This may be caused by the tool’s geometry, which does not promote good chip removal, potentially leading to poorer cutting performance in the final stage. This may result in greater compressive forces and contribute to greater delamination of the composite. When focusing exclusively on tests that used the same feed per tooth with the BZT03 tool, the findings suggest that increasing drilling speed reduces damage at the hole’s exit, despite the associated rise in temperature.

When comparing the tools, the BZT03 tool exhibits a wider range of results in contrast to the Dagger. With the Dagger tool, the exit-adjusted delamination factor remains consistently lower than with the BZT03, regardless of spindle speed. Additionally, while maximum temperature increases at higher spindle speeds, these temperatures are notably lower than those observed when using the BZT03 tool. The Dagger tool demonstrates an ability to improve damage at the hole exit while simultaneously reducing temperature. However, establishing strong correlations between these two variables remains challenging.

## Figures and Tables

**Table 1:** Plate characterization.

Reinforcement	Number of Layers	Thickness (mm)	Glass Fiber
Fabric (long fiber)	10	2.88	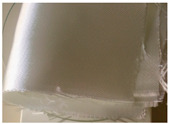
	10	3.00	
	10	2.95	
	20	5.54	
	30	8.36	
Mat (short fiber)	10	6.37	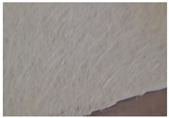

**Table 2:** Description of input and output variables.

**Input Variables**	**Data**
Tool	BZT01, BZT03, Dagger, Seco
Spindle speed (rpm)	1300, 2650, 5300
Feed rate (mm/min)	78, 156, 234, 318, 640
Thickness (mm)	2.88, 2.95, 3, 5.54, 8.36
**Output variables**	**Acronym**
Exit delamination factor	Fd_out
Exit delaminated area factor	Fda_out
Exit-adjusted delamination factor	Fdaj_out
Exit delaminated area	Area_out
Exit maximum temperature	Tmax_inf

**Table 3:** Experiment characteristics for output variables assessment using the tools BZT01, BZT03, Seco, and Dagger.

Tool	Thickness (mm)	Spindle Speed (rpm)	Feed Rate (mm/min)	Number of Tests
BZT01	2.88 (low)	5300	640	5
Seco	2.88 (low)	5300	640	5
BZT03	2.88 (low)	5300	640	10
	5.54 (medium)	5300	640	5
	8.36 (high)	5300	640	3
	3 (low)	2650	318	4
	3 (low)	1300	156	5
	3 (low)	1300	78	5
	3 (low)	1300	234	5
Dagger	2.88 (low)	5300	640	9
	2.95 (low)	2650	318	5
	2.95 (low)	1300	156	5
	2.95 (low)	1300	78	5
	2.95 (low)	1300	234	5

**Table 4:** ANOVA results from the comparison of the Fadj_out mean values for the four tools: BZT 01, BZT 03, Dagger, and Seco.

	Degrees of Freedom	Sum of Squares	Mean Squares	F	*p*-Value ^1^
**Tool**	3	42.87	14.29	16.38	<0.001 **
**Residuals**	16	13.96	0.87		

^1^ Significance codes: <0.001 ‘**’ 0.01 ‘*’.

**Table 5:** Experiment characteristics for exit-adjusted delamination factor assessment using BZT03 and Dagger tools.

Tool	Thickness (mm)	Spindle Speed (rpm)	Feed Rate (mm/min)	Number of Tests
BZT 03	2.88	5300	640	10
	3	2650	318	4
	3	1300	156	5
	3	1300	78	5
	3	1300	234	5
Dagger	2.88	5300	640	9
	2.95	2650	318	5
	2.95	1300	156	5
	2.95	1300	78	5
	2.95	1300	234	5

**Table 6:** Regarding the BZT 03 tool, ANOVA results from the comparison of the Fadj_out mean values for the three ranges of the spindle speed.

BZT03	Degrees of Freedom	Sum of Squares	Mean Squares	F	*p*-Value ^1^
**Spindle**	2	0.10	0.07	0.01	0.99
**Residuals**	34	327.30	9.63		

^1^ Significance codes: <0.001 ‘**’ 0.01 ‘*’.

**Table 7:** Regarding the Dagger tool, ANOVA results from the comparison of the Fadj_out mean values for the three ranges of the spindle speed.

Dagger	Degrees of Freedom	Sum of Squares	Mean Squares	F	*p*-Value ^1^
**Spindle**	2	0.15	0.07	4.27	0.02
**Residuals**	26	0.45	0.02		

^1^ Significance codes: <0.001 ‘**’ 0.01 ‘*’.

**Table 8:** Regarding the BZT 03 tool, ANOVA results from the comparison of the Fadj_out mean values for the five ranges of the feed rate.

BZT03	Degrees of Freedom	Sum of Squares	Mean Squares	F	*p*-Value ^1^
**Feed rate**	4	62.35	15.59	1.88	0.14
**Residuals**	32	265.11	8.285		

^1^ Significance codes: <0.001 ‘**’ 0.01 ‘*’.

**Table 9:** Regarding the Dagger tool, ANOVA results from the comparison of the Fadj_out mean values for the five ranges of the feed rate.

Dagger	Degrees of Freedom	Sum of Squares	Mean Squares	F	*p*-Value ^1^
**Feed rate**	4	0.16	0.04	2.22	0.10
**Residuals**	24	0.44	0.02		

^1^ Significance codes: <0.001 ‘**’ 0.01 ‘*’.

**Table 10:** Regarding the BZT03 tool, ANOVA results from the comparison of the Tmax_inf mean values for the three ranges of the spindle speed.

BZT03	Degrees of Freedom	Sum of Squares	Mean Squares	F	*p*-Value ^1^
**Spindle**	2	21,371	10,685	4.11	0.03
**Residuals**	33	85,827	2601		

^1^ Significance codes: <0.001 ‘**’ 0.01 ‘*’.

**Table 11:** Regarding the Dagger tool, ANOVA results from the comparison of the Tmax_inf mean values for the three ranges of the spindle speed.

Dagger	Degrees of Freedom	Sum of Squares	Mean Squares	F	*p*-Value ^1^
**Spindle**	2	4347	2173.6	21.57	<0.001 **
**Residuals**	26	2620	100.8		

^1^ Significance codes: <0.001 ‘**’ 0.01 ‘*’.

**Table 12:** Regarding the Dagger tool, ANOVA results from the comparison of the Tmax_inf mean values for the five ranges of the feed rate.

Dagger	Degrees of Freedom	Sum of Squares	Mean Squares	F	*p*-Value ^1^
**Spindle**	4	4755	1188.7	12.89	<0.001 **
**Residuals**	24	2213	92.2		

^1^ Significance codes: <0.001 ‘**’ 0.01 ‘*’.

**Table 13:** Set of experimental tests with the BZT03 tool for the maximum temperature at the tool’s exit.

Thickness (mm)	Spindle Speed (rpm)	Feed Rate (mm/min)	Number of Tests
2.88	5300	640	10
5.54	5300	640	5
8.36	5300	640	3
3	2650	318	4
3	1300	156	5
3	1300	78	5
3	1300	234	5

**Table 14:** ANOVA results from the comparison of the Fdaj_out mean values for the three ranges of thickness.

BZT03	Degrees of Freedom	Sum of Squares	Mean Squares	F	*p*-Value ^1^
**Thickness**	2	159.7	79.83	16.17	<0.001**
**Residuals**	34	167.8	4.94		

^1^ Significance codes: <0.001 ‘**’ 0.01 ‘*’.

## Data Availability

The original contributions presented in the study are included in the article, further inquiries can be directed to the corresponding author.

## Appendix A

**Table:**

Tool	Thickness [mm]	Spindle Speed (rpm)	Feed Rate [mm/min]	Fdaj_out	Tmax_inf
BZT01	2.88 (low)	5300	640	3.595	250
				3.481	250
				3.699	250
				3.769	241.638
				3.277	233.044
Seco	2.88 (low)	5300	640	1.434	79.116
				1.802	69.268
				1.933	75.524
				1.818	87.314
				2.026	86.646
				1.434	79.116
BZT03	2.88 (low)	5300	640	3.647	242.387
				3.653	250
				3.395	250
				4.199	248.443
				3.921	236.274
				4.385	222.716
				5.529	214.491
				7.907	229.016
				2.988	223.528
				4.340	224.933
	5.54 (medium)	5300	640	5.592	119.338
				9.636	122
				9.544	88
				7.407	121
				5.008	121
	8.36 (high)	5300	640	11.436	87.982
				15.935	88
				12.956	88
	3 (low)	2650	318	6.935	232.101
				6.819	246.467
				7.527	237.575
				5.316	225.816
		1300	156	5.896	225.668
				8.028	-----
				9.689	227.145
				5.207	242.438
				5.556	219.759
			78	4.081	243.046
				4.623	228.579
				3.914	246
				3.522	246
				3.908	246
			234	6.882	195.256
				8.365	209.159
				9.493	192.476
				9.586	204.38
				10.567	173.591
Dagger	2.88 (low)	5300	640	1.415	150.771
				1.319	133.857
				1.235	140.142
				1.416	139.302
				1.371	142.372
				1.415	150.771
				1.319	133.857
				1.235	140.142
				1.416	139.302
	2.95 (low)	2650	318	1.368	115.851
				1.308	121.286
				1.238	114.886
				1.323	117.232
				1.440	112.424
		1300	156	1.347	125.457
				1.338	101.128
				1.591	110.194
				1.449	122.96
				1.435	141.387
			78	1.655	110.338
				1.474	106.805
				1.644	118.796
				1.242	123.43
				1.411	140.866
			234	1.247	114.181
				1.494	106.892
				1.874	108.239
				1.389	103.857
				1.509	112.236
